# Longer Work/Rest Intervals During High-Intensity Interval Training (HIIT) Lead to Elevated Levels of miR-222 and miR-29c

**DOI:** 10.3389/fphys.2018.00395

**Published:** 2018-04-17

**Authors:** Boris Schmitz, Florian Rolfes, Katrin Schelleckes, Mirja Mewes, Lothar Thorwesten, Michael Krüger, Andreas Klose, Stefan-Martin Brand

**Affiliations:** ^1^Institute of Sports Medicine, Molecular Genetics of Cardiovascular Disease, University Hospital Muenster, Muenster, Germany; ^2^Internal Medicine D, Nephrology, Hypertension and Rheumatology, University Hospital Muenster, Muenster, Germany; ^3^Department of Physical Education and Sports History, University of Muenster, Muenster, Germany

**Keywords:** microRNA, recovery, performance, sprint interval training (SIT), high intensity training (HIT)

## Abstract

**Aim:** MicroRNA-222 (miR-222) and miR-29c have been identified as important modulators of cardiac growth and may protect against pathological cardiac remodeling. miR-222 and -29c may thus serve as functional biomarkers for exercise-induced cardiac adaptations. This investigation compared the effect of two workload-matched high-intensity interval training (HIIT) protocols with different recovery periods on miR-222 and -29c levels.

**Methods:** Sixty-three moderately trained females and males (22.0 ± 1.7 years) fulfilled the eligibility criteria and were randomized into two HIIT groups using sex and exercise capacity. During a controlled 4-week intervention (two sessions/week) a 4 × 30 HIIT group performed 4 × 30 s runs (all-out, 30 s active recovery) and a 8 × 15 HIIT group performed 8 × 15 s runs (all-out, 15 s active recovery). miR-222 and -29c as well as transforming growth factor-beta1 (TGF-beta1) mRNA levels were determined during high-intensity running as well as aerobic exercise using capillary blood from earlobes. Performance parameters were assessed using an incremental continuous running test (ICRT) protocol with blood lactate diagnostic and heart rate (HR) monitoring to determine HR recovery and power output at individual anaerobic threshold (IAT).

**Results:** At baseline, acute exercise miR-222 and -29c levels were increased only in the 4 × 30 HIIT group (both *p* < 0.01, pre- vs. post-exercise). After the intervention, acute exercise miR-222 levels were still increased in the 4 × 30 HIIT group (*p* < 0.01, pre- vs. post-exercise) while in the 8 × 15 HIIT group again no acute effect was observed. However, both HIIT interventions resulted in elevated resting miR-222 and -29c levels (all *p* < 0.001, pre- vs. post-intervention). Neither of the two miRNAs were elevated at any ICRT speed level at baseline nor follow-up. While HR recovery was improved by >24% in both HIIT groups (both *p* ≤ 0.0002) speed at IAT was improved by 3.6% only in the 4 × 30 HIIT group (*p* < 0.0132). Correlation analysis suggested an association between both miRNAs and TGF-beta1 mRNA (all *p* ≤ 0.006, *r* ≥ 0.74) as well as change in speed at IAT and change in miR-222 levels (*p* = 0.024, *r* = 0.46).

**Conclusions:** HIIT can induce increased circulating levels of cardiac growth-associated miR-222 and -29c. miR-222 and miR-29c could be useful markers to monitor HIIT response in general and to identify optimal work/rest combinations.

## Introduction

High-intensity interval training (HIIT) has become a well-established training component of athletes to improve aerobic endurance and maximal exercise capacity (Sloth et al., 2013; Weston et al., 2014; Milanović et al., 2015). In addition, HIIT has been suggested as efficient tool to improve health-related fitness in the general population (Burgomaster et al., 2008; Weston et al., 2014; Costigan et al., 2015) and the prevention of lifestyle-induced chronic diseases such as type 2 diabetes mellitus, arterial hypertension, obesity and the metabolic syndrome (Weston et al., 2014) as well as coronary artery disease and heart failure (Guiraud et al., 2012; Ellingsen et al., 2017). Compared to endurance training, HIIT is marked by brief bursts of near-maximal to supra-maximal work rates followed by short periods of rest or active recovery accompanied by an overall reduction in training duration (Burgomaster et al., 2008; Milanović et al., 2015). While the overall HIIT effects in terms of sub-maximal and maximal performance have recently been analyzed in some detail (Weston et al., 2014; Costigan et al., 2015; Milanović et al., 2015; Liou et al., 2016), organ-specific and cellular effects of HIIT and the underlying molecular mechanisms are still incompletely understood. In terms of cardiac adaptations, recent investigations suggested that metabolic and functional changes in the heart are already detectable after 2 weeks of HIIT (six exercise sessions, 4 – 6 × 30 s of all-out cycling) (Eskelinen et al., 2016; Heiskanen et al., 2016). Stöggl and Björklund (2017) analyzed the cardiovascular response in terms of acute HR recovery to a 9-week HIIT intervention in endurance athletes (27 exercise sessions, 4 × 4 min at > 90% HR_max_ running or cycling) and found that HR recovery was improved by 11.2%. Notably, HIIT has also been shown to cause cardiac adaptations in clinical settings. A 12-week HIIT (4 × 4 min at > 90% HR_max_, 3 times/week, uphill walking) performed by patients with stable post-infarction heart failure induced reverse remodeling of the left ventricle (Wisløff et al., 2007).

MicroRNAs (miRNAs) have been identified as potent markers for training adaptations and individual exercise response (Zacharewicz et al., 2013; Flowers et al., 2015; Polakovičová et al., 2016). Moreover, well-characterized miRNAs may have the potential to serve as functional biomarkers and reveal physiological processes involved in the response to specific training intervention (Schmitz et al., 2017b). miRNAs are short (~21 nucleotide-long) endogenous non-coding RNAs involved in translational repression (Filipowicz et al., 2008; Huntzinger and Izaurralde, 2011). Essential functions of miRNAs have been identified in almost every physiological process including development, aging and disease (Alvarez-Garcia and Miska, 2005; Sayed and Abdellatif, 2011; Jung and Suh, 2014). Following the identification of muscle-specific miRNAs and their role in skeletal muscle development, plasticity and regeneration (McCarthy and Esser, 2007; Simionescu-Bankston and Kumar, 2016), the discovery of inducible circulating plasma miRNAs has set the stage to monitor the physiological response already during exercise (Wehmeier and Hilberg, 2014; Schmitz et al., 2017b). Of note, selectively released plasma miRNAs are preserved by association with RNA-binding proteins or small membranous vesicles and commonly involved in inter-cell communication (Deregibus et al., 2007; Arroyo et al., 2011).

With respect to cardiac adaptations, miR-222 and miR-29c have recently been identified as important modulators. Liu et al. (2015) identified miR-222 upregulation in mice subjected to two different 3-week exercise protocols (running or swimming). Subsequent studies revealed that miR-222 increased markers of cardiomyocyte proliferation and the group was able to show that miR-222 was necessary for exercise-induced cardiac growth and protected against cardiac remodeling and dysfunction after ischemic injury (Liu et al., 2015). Members of the miR-29 family have also been identified as important modulator of cardiac remodeling in post-myocardial infarction mice (van Rooij et al., 2008). Further analyzes suggested that miR-29 may regulate multiple gene expression programs in the heart and that strategies to enhance miR-29 levels may have therapeutic value (van Rooij et al., 2008). Since TGF-β signaling plays a central role in the regulation of hypertrophic processes (Rao et al., 2011; Wang and Yang, 2012), it is also of interest that the TGF-β pathway has been suggested as a target of miR-222 and -29c (Rao et al., 2011; Wardle et al., 2015; Zhang et al., 2016). These observations suggest miR-222 and miR-29 as functional biomarkers for exercise-induced cardiac adaptations.

The current investigation aimed to characterize weather circulating levels of miR-222 and miR-29c can be upregulated by all-out running HIIT. Therefore we analyzed acute changes of both miRNAs in response to a single training session at baseline and follow-up as well as the effect of a 4-week HIIT intervention on resting miRNA levels. In addition, we investigated which exercise intensities are sufficient to induce effects on miR-222 and -29c levels during a continuous incremental running protocol. A second objective of our study was to examine if a 4 × 30 HIIT intervention would induce different training adaptations in comparison to a time and workload matched 8 × 15 HIIT protocol. We hypothesized that longer work/ rest intervals of the 4 × 30 protocol would induce greater improvement of performance parameters and stronger effects on miR-222 and -29c levels.

## Methods

### Study design

**Figure 1:**
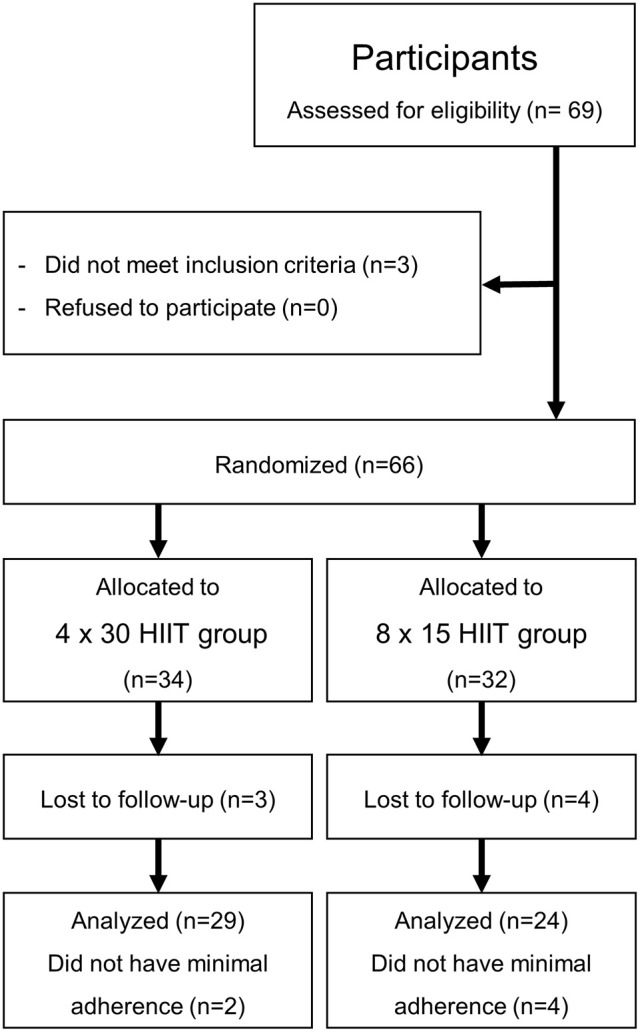
Study flow chart.

A randomized controlled interventional study design was used to compare the effects of two different 4-week HIIT programs in young healthy moderately trained individuals on circulating miR-222 and miR-29c levels at rest and post-exercise during baseline and follow-up high-intensity running (Figure 1). Additional samples for miRNA determination were drawn during baseline and follow-up continuous running exercise. We selected a 4 × 30 HIIT protocol and a time- and workload-matched 8 × 15 HIIT protocol since it has been suggested that work/rest intervals of 30/30 s could result in smaller disruption of the post-exercise cardiac autonomic modulation compared with shorter 15 s work/rest intervals (Cipryan et al., 2016). Training effects on aerobic performance and exercise capacity were assessed as power output (speed) at individual anaerobic threshold (IAT) defined as baseline LA + 1.5 mmol/l (1.5 mmol/l above lactate equivalent; Roecker et al., 1998; Dickhuth et al., 1999) and maximal running speed. HR recovery defined as HR_max_ - HR_3min_ was accessed as parameter for the cardiovascular response to the intervention (Okutucu et al., 2011; Stöggl and Björklund, 2017).

### Participants

Initially, 69 young healthy moderately trained female and male students of the University's Physical Education Department were recruited at the Institute of Sports Medicine of the University Hospital Muenster in May 2017 (Figure 1). All investigations were performed in accordance with the declaration of Helsinki and after the approval of the local ethics committee of the medical association Westfalen-Lippe and the Westphalian Wilhelms-University of Muenster (project-no. 2013-231-f-S, study acronym SPORTIVA). Written informed consent of participants was obtained prior to subjects' participation in the study. Inclusion criteria were age >18 years, a health certificate as necessary to study at the University's Physical Education Department and valid baseline maximal performance exercise tests (see below). Participants were randomized to either one of the training groups using exercise capacity determined by a standardized incremental continuous running test (ICRT). The sample size was calculated based on a previous investigation of HIIT on performance with a detected effect size (ES; Cohen's d) = 0.5 revealing that a sample size of 27 per group would yield a statistical power of 1-beta = 0.8 at alpha = 0.05. Sixty-six students fulfilled the inclusion criteria, three students had an incomplete baseline exercise test. Exclusion criteria were injury/ illness during the training period and missing adherence to the training program. In total, 13 participants dropped out of the study (Figure 1). Participants were involved in different activities outside the study protocol (documented in training logs) such as team sports, resistance training, etc. which had already been performed prior to the training intervention and were distributed equally over the two training groups (4 × 30 group, 296.0 ± 185.5 min/week; 8 × 15 group, 299.0 ± 192.3 min/week; *p* = 0.9625). Participants' diet was not controlled.

### Test procedures

During the 1st week, participants' anthropometric data was recorded using standard medical equipment (Table 1) and participants were familiarized with the study protocol and test procedures. During the 2nd week, all participants performed a standardized ICRT to determine baseline individual lactate thresholds, HR recovery and exercise capacity. In the 3rd week, participants performed the first HIIT sessions of the 4-week intervention. The follow-up ICRT was performed 7 days after the last training session at identical daytime.

**Table 1:** Participants' anthropometric data at baseline.

	**4 × 30 (*n* = 29)**	**8 × 15 (*n* = 24)**	***P*-value**
Female, n	21 (72.4)	18 (75.0)	0.836
Age, yrs	21.9 ± 1.7	22.2 ± 1.8	0.492
Height, cm	174.2 ± 7.3	174.3 ± 7.3	0.969
Body mass, kg	68.5 ± 10.7	66.3 ± 10.6	0.461
BMI, kg·m^−2^	22.5 ± 2.5	21.7 ± 2.0	0.219

*Data are means ± SD or n (%). BMI, body mass index*.

#### Exercise test procedures

To assess exercise parameters at baseline and follow-up, the ICRT field test was performed as described elsewhere (Léger and Boucher, 1980; Berthoin et al., 1994; McGehee et al., 2005) with modifications (Schmitz et al., 2017a). The test was performed indoors on a synthetic 200 m running track at ambient temperature (18–22°C, ~60 m above sea level) in groups of 4 individuals and a minimum of 4 assistants and 2 trainers. Subjects were fitted with HR monitors combined with a wireless receiver module (Acentas, Muenster, Germany) to determine exercise HR with up to 300 s of recording after the test to assess HR during passive recovery (standing). HR recovery was calculated from delta HR_max_ - HR_3min_. The test started at 2.22 m·s^−1^ (no separate warm-up included), increasing by 0.56 m·s^−1^ every 3 min until total exhaustion of the participant. The pace was indicated by an automated acoustic device. Blood was sampled from participants' earlobes for blood lactate concentration measurement (20 μl heparinized capillary; automated on analyzer Biosen S-line, EKF Diagnostics, Magdeburg, Germany) and miRNA measurement (20 μl K_2_ EDTA capillary) before the test, after each interval (3 min) and at 3 min and 5 min after the test. Power output at IAT (baseline lactate + 1.5 mmol·L^−1^) (Roecker et al., 1998; Dickhuth et al., 1999) was calculated using Winlactat software version 5.0.0.54 (Mesics, Muenster, Germany). Participants were asked to give their rating of perceived exertion (RPE) using the 6–20 Borg scale (Borg, 1982) after each 3 min interval.

### HIIT interventions

The study included two training groups which were matched for workload and total training duration (Table 2). During the 4-week training intervention the participants performed two controlled exercise sessions/week (1 day off between sessions) supervised by at least one experienced trainer. All training sessions started with a warm-up phase of 10 min including running at 2.22 m·s^−1^ and light stretching. A cool-down phase was not included.

1. 4 × 30 HIIT: participants were instructed to run at maximal speed for 4 × 30 s (all-out) with 30 s of active recovery periods at warm-up speed between bouts.
2. 8 × 15 HIIT: participants were instructed to run at maximal speed for 8 × 15 s (all-out) with 15 s of active recovery periods at warm-up speed between bouts.

**Table 2:** HIIT workload per group.

	**4 × 30 HIIT (two sessions/week)**		**8 × 15 HIIT (two sessions/week)**	
	**30 s runs**	**MET·min·wk^−1^**	**15 s runs**	**MET·min·wk^−1^**
Week 1	8	260	16	260
Week 2	8	260	16	260
Week 3	8	260	16	260
Week 4	8	260	16	260
Total	32	1,040	64	1,040

*HIIT, high-intensity interval training; MET, metabolic equivalent. METs were estimated according to the Compendium of Physical Activities (Ainsworth et al., 2000) with 8.0 METs for running at 2.2 m·s^−1^ (code 12030) and 19.0 METs for running at 5.5 m·s^−1^ (code 12132). METs for both HIIT groups include warm-up (10 min at ~ 2.2 m·s^−1^) and active recovery phases (30/15 s at ~2.2 m·s^−1^)*.

The two HIIT interventions were designed with active (~ 2.2 m·s^−1^) but only 30/15 s of recovery that would not allow total recovery within each training set. Respective metabolic equivalents (METs) were estimated according to the Compendium of Physical Activities (Ainsworth et al., 2000) with 8.0 METs for running at 2.2 m·s^−1^ (code 12030) and 19.0 METs for running at 5.5 m·s^−1^ (code 12132) as reported previously (Schmitz et al., 2017b). Additional blood sampling for miRNA quantification was performed during the first (baseline) and the last (post-training) HIIT sessions at rest and post-exercise. Data are presented using the nomenclature suggested by Baggish et al. (2011).

### miRNA extraction and quantification

Blood sampling from participants' earlobes was performed as reported previously (Wehmeier and Hilberg, 2014; Kilian et al., 2016; Schmitz et al., 2017b) immediately at the testing site using a 20 μl K_2_ EDTA capillary (Sarstedt, Nuernbrecht, Germany) and RNA was extracted using 750 μl peqGOLD TriFast (VWR, Darmstadt, Germany) according to the manufacturer's instruction. Each sample was immediately supplemented with 10 nM *Caenorhabditis elegans* cel-miR-39-3p spike-in control following manufacturer's instruction (Thermo Fisher Scientific, Darmstadt, Germany) for normalization (Fichtlscherer et al., 2010; Schlosser et al., 2015; Schmitz et al., 2017b). RNase-free glycogen (70 μg/sample; VWR) was used as carrier to optimize extraction efficiency (McAlexander et al., 2013). Isolated RNA was resuspended in 20 μl of nuclease-free water. Quantification of mature hsa-miR-222-3p, hsa-miR-29c-3p and cel-miR-39-3p as well as Transforming Growth Factor-beta 1 (TGF-β1) mRNA was performed by quantitative real-time polymerase chain reaction (qRT-PCR) using 5′ adaptor ligation and target-independent cDNA generation in a single reaction (TaqMan Advanced MicroRNA technology; Thermo Fisher Scientific, Darmstadt, Germany). In brief, 1.0 μl of RNA solution was used for adaptor ligation and reverse transcription according to manufacturer's instructions. cDNA was diluted 1:10 in ultra-pure water und 1.25 μl were used for final qRT-PCR reactions performed in a 384-well format in duplicates on an ABI7500 fast RT-PCR system (Life Technologies, Carlsbad, USA). Relative quantification was performed using the ΔCt method and miR-222 and miR-29c values were expressed as (1/ΔCt)*100 for presentation. Duplicates with a difference greater than 1 Ct were excluded from the analysis. Samples for miRNA analysis were available of 28 participants from the 4 × 30 HIIT group and 23 participants of the 8 × 15 HIIT group. Five male participants from both HIIT groups were randomly selected to analyze miRNA changes during ICRT at baseline and follow-up (including 3 and 5 min of recovery).

### Statistical data analysis

Statistical analyses were performed using SPSS, version 23.0 (Statistical Package for Social Science, Chicago, USA) and GraphPad PRISM V5.0 software (GraphPad Software Inc., La Jolla, USA). Data are presented as mean ± SD or 95% confidence interval (CI) where indicated. Differences were determined using paired two-sided *t*-test or ANOVA with *post-hoc* correction (Kruskal-Wallis test with Dunn's *post-hoc* analysis) as indicated. Data were tested for normal distribution using D'Agostino-Pearson normality test (omnibus K2 test). Correlation between changes in IAT, mRNA and miRNA levels were analyzed using Spearman's rank test. Significance was declared at *p* < 0.05. The magnitudes of changes after training were expressed as standardized effect size (ES), calculated from means and SD (Cohen's d). Power calculations were performed using G^*^Power 3.1.9.2.

## Results

### HIIT effects on exercise performance parameters

Participants' ICRT exercise data before and after the 4-week training intervention are presented in Table 3. No significant difference existed between the two HIIT groups at baseline (all *p* > 0.39). A significant increase in speed at IAT during ICRT re-test was detected for the 4 × 30 HIIT group in comparison to the 8 × 15 HIIT group which showed no effect on speed at IAT (between-group *p* < 0.0132, before vs. after training). Consistently, 8 × 15 HIIT participants reached the IAT at follow-up at lower HR compared to the 4 × 30 HIIT group (between-group *p* < 0.0232, before vs. after training). Maximal exercise capacity (maximal speed) during ICRT re-test was significantly increased in both HIIT groups (both *p* < 0.05, before vs. after training). While maximal HR was not significantly different between baseline and follow-up (both *p* > 0.195, before vs. after training), a strong improvement of HR recovery (HR_max_ - HR_3min_) during ICRT re-test was detected for both HIIT groups (both *p* ≤ 0.0002, before vs. after training).

**Table 3:** Participants' exercise performance parameters and pre- vs. post-changes.

**Variable**	**Test**	**4 × 30 (*n* = 29)**	**Effect size**	**8 × 15 (*n* = 24)**	**Effect size**	***P*-value 4 × 30 vs. 8 × 15 baseline**	***P*-value 4 × 30 vs. 8 × 15 pre-post**
Resting HR, beats·min^−1^	Pre	100.4 ± 14.7	−0.22	96.3 ± 19.8	0.13	0.5989	0.3334
	Post	97.0 ± 15.9		98.8 ± 21.0			
	Δ%	−3.39		+ 2.67			
	*p*-value	0.3717		0.9905			
Resting LA, mmol·L^−1^	Pre	1.3 ± 0.4	−0.25	1.3 ± 0.5	−0.09	0.7086	0.8399
	Post	1.2 ± 0.4		1.3 ± 0.4			
	Δ%	−7.32		−2.92			
	*p*-value	0.3638		0.5067			
Speed at IAT, m·s^−1^	Pre	3.00 ± 0.33	0.34	3.06 ± 0.30	−0.12	0.5362	**0.0132^#^**
	Post	3.11 ± 0.30		3.02 ± 0.30			
	Δ%	+ 3.6^*^		−1.43			
	*p*-value	**0.0116**		0.3190			
HR at IAT, beats·min^−1^	Pre	175.3 ± 9.8	0.11	175.0 ± 12.5	−0.19	0.9087	**0.0232^#^**
	Post	176.3 ± 8.2		172.8 ± 10.0			
	Δ%	+ 0.57		−1.26^*^			
	*p*-value	0.3873		**0.0346**			
Maximal speed, m·s^−1^	Pre	4.25 ± 0.55	0.28	4.37 ± 0.51	0.21	0.3935	0.9332
	Post	4.39 ± 0.48		4.48 ± 0.52			
	Δ%	+ 3.37^*^		+ 2.47^*^			
	*p*-value	**0.0048**		**0.0415**			
Maximal LA, mmol·L^−1^	Pre	12.7 ± 2.5	0.0	12.9 ± 2.9	−0.08	0.7918	0.8322
	Post	12.7 ± 2.6		12.6 ± 3.3			
	Δ%	± 0.0		−2.3			
	*p*-value	0.9050		0.9447			
Maximal RPE, Borg scale	Pre	19.9 ± 0.2	0.22	19.9 ± 0.2	0.27	0.8939	0.8322
	Post	20.0 ± 0.0		20.0 ± 0.0			
	Δ%	+ 0.15		+ 0.15			
	*p*-value	0.3265		0.3299			
Maximal HR, beats·min^−1^	Pre	196.4 ± 7.8	0.13	197.8 ± 8.9	−0.05	0.5595	0.1161
	Post	197.4 ± 7.5		197.4 ± 5.4			
	Δ%	+ 0.51		−0.20			
	*p*-value	0.4019		0.1949			
HR recovery, beats·min^−1^	Pre	−59.0 ± 15.2	1.14	−55.7 ± 13.1	1.37	0.4017	0.5277
	Post	−73.6 ± 9.7		−72.8 ± 11.5			
	Δ%	+ 24.8^*^		+ 30.7^*^			
	*p*-value	**<0.0001**		**0.0002**			

*Data presented as mean ± SD. Changes (Δ) are presented in percent. Group 4 × 30, high-intensity interval training with 4 runs of 30 s duration; Group 8 × 15, high-intensity interval training with 8 runs of 15 s duration; HR, heart rate; HR recovery, HR_max_ - HR_3min_; LA, blood lactate concentration; IAT, individual anaerobic threshold (baseline lactate + 1.5 mmol·L^−1^); resting HR, HR determined before start of the exercise test; RPE, rated perceived exertion*.

* *Significantly different from pre-intervention by within-group two-tailed paired t-test*.

# *Significantly different by between-group two-way ANOVA. Effect sizes were calculated from means and SD (Cohen's d). All significant p-values have been highlighted*.

### HIIT effects on acute exercise miR-222 and -29c levels

**Figure 2:**
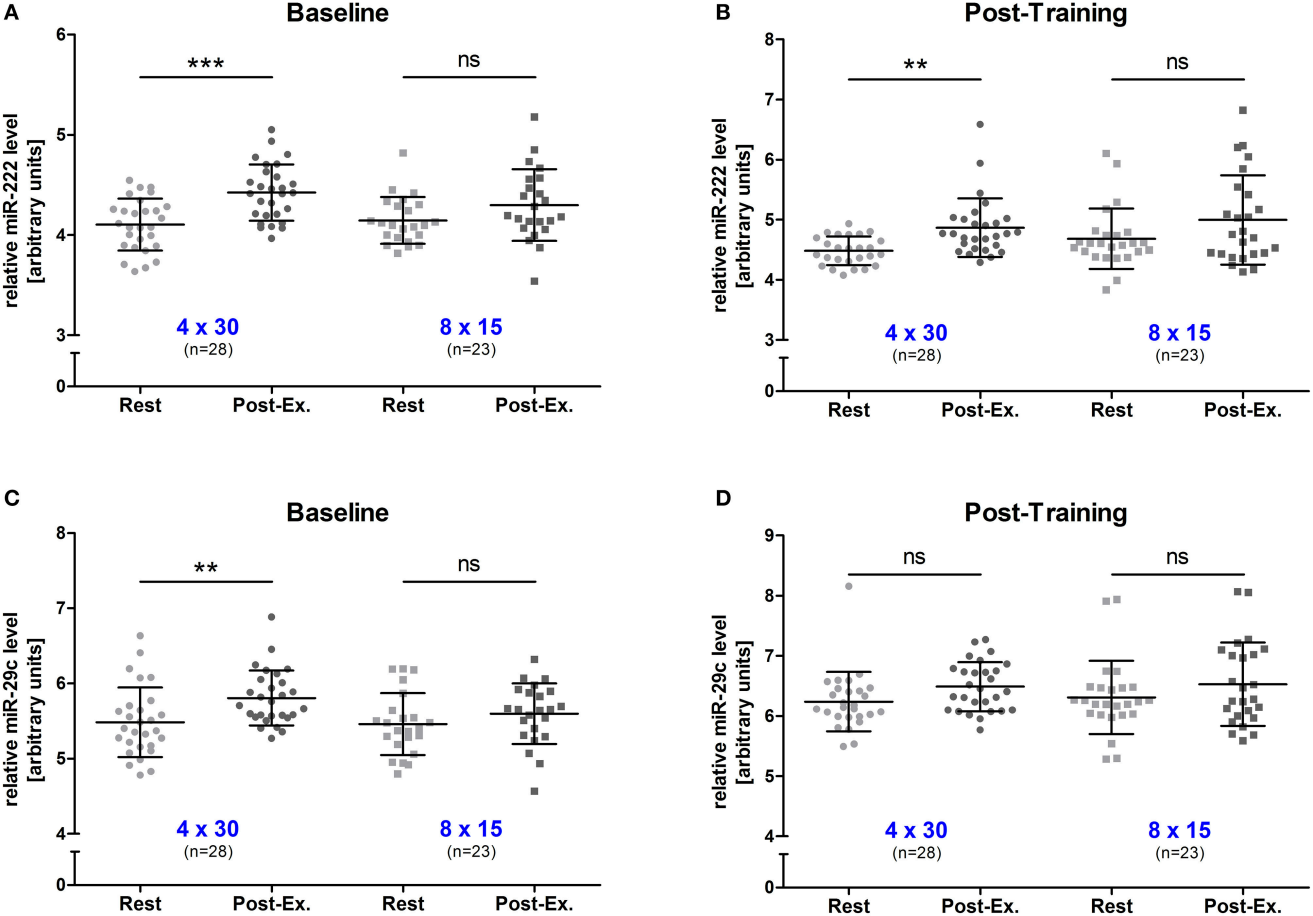
Exercise miR-222 and miR-29c levels were increased in the 4 × 30 HIIT group but not the 8 × 15 HIIT group. The acute effect of a single exercise session before the intervention was determined for **(A)** miR-222 and **(C)** miR-29c levels. A significant increase was only detected in the 4 × 30 HIIT group. At follow-up, the acute exercise effect on **(B)** miR-222 was still detectable while no increase was seen for **(D)** miR-29c. Again, no effect was detected within the 8 × 15 HIIT group. HIIT participants were tested directly before and after all-out high-intensity runs. Each participant is represented by one data point. Data are represented as mean ± SD. *P*-values are rest vs. post-exercise using ANOVA. ^**^p < 0.01; ^***^p < 0.001; ns, not significant.

To determine changes in miR-222 and -29c levels in response to an acute high-intensity exercise bout, blood samples were drawn immediately before (rest) and directly after (post-exercise) the first training session (baseline) and the last training session (post-training). At baseline, we detected that 4 × 30 s of high-intensity running induced a significant increase in both miRNAs (both *p* < 0.01, rest vs. post-exercise) while neither of the two miRNAs was elevated in the 8 × 15 HIIT group (both *p* > 0.05, rest vs. post-exercise; Figures 2A,C). After the 4-week training period, a significant acute increase for miR-222 was detected (*p* < 0.01, rest vs. post-exercise) in the 4 × 30 HIIT group but miR-29c levels remained unchanged (*p* > 0.05, rest vs. post-exercise). Again, no acute exercise effect on miRNA levels was seen for the 8 × 15 HIIT group (both *p* > 0.05, rest vs. post-exercise; Figures 2B,D). These data indicate that despite the time and workload matched exercise, different acute physiological responses might exist for the two HIIT protocols. We also analyzed miR-222 and -29c levels for a potential effect by sex and detected no difference between male and female participants for the two miRNAs at baseline (both *p* > 0.05, rest vs. post-exercise; Supplemental Figure 1). However, we observed a trend toward stronger acute effects on miR-222 levels in male participants at follow-up (*p* = 0.066, males + 0.735 ± 0.517 units vs. females + 0.204 ± 0.970 units, rest vs. post-exercise).

### HIIT effects on resting miR-222,-29c and TGF-β1 mRNA levels

**Figure 3:**
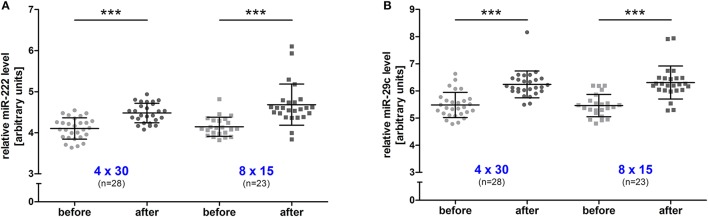
Resting miR-222 and miR-29c levels were increased in both HIIT groups after the intervention. After the 4-week training intervention, a significant increase of resting levels was detected for **(A)** miR-222 and **(B)** miR-29c in the 4 × 30 HIIT and the 8 × 15 HIIT group. HIIT participants were tested directly before all-out high-intensity runs at baseline (before) and follow-up (after). Each participant is represented by one data point. Data are represented as mean ± SD. *P*-values are before vs. after intervention using ANOVA. ^***^p < 0.001.

**Figure 4:**
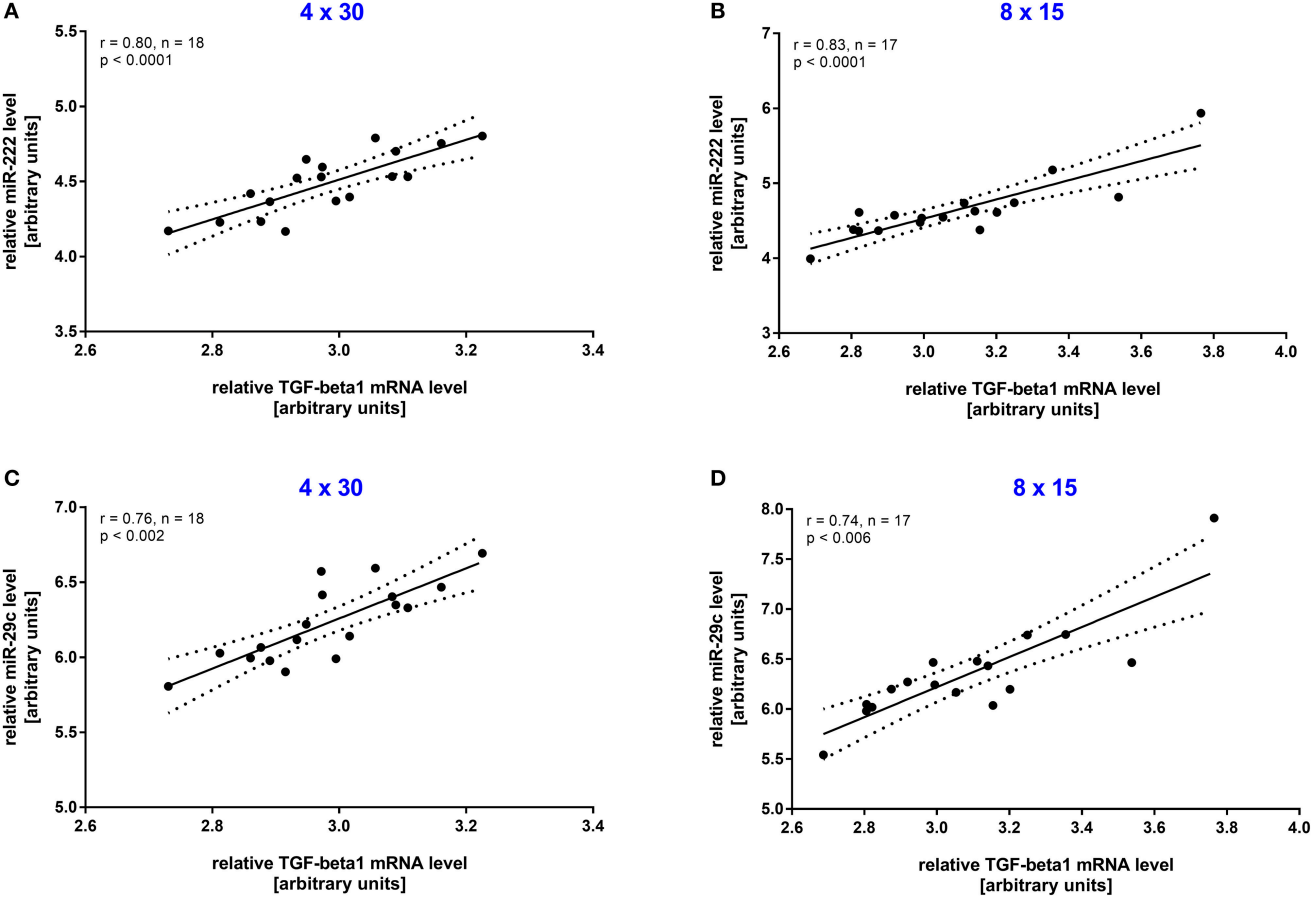
Resting miR-222 and miR-29c levels were associated with TGF-β1 mRNA levels after the intervention. After the 4-week training intervention a significant correlation in resting TGF-β1 mRNA levels and miR-222 and miR-29s levels was detected in the **(A,C)** 4 × 30 HIIT group as well as the **(B,D)** 8 × 15 HIIT group. Individual data points are shown with 95% CI and linear regression.

To investigate the long-term effect of HIIT, resting blood samples drawn immediately before the first and the last training session were compared. A significant increase (all *p* < 0.001, before vs. after training) was detected for both miRNAs in the 4 × 30 HIIT group (Figure 3A). Of note, the 8 × 15 HIIT group also presented a significant increase (both *p* < 0.001, before vs. after training) of resting miRNA levels (Figure 3B). A between-group analysis did not reveal differences in resting miRNA levels between the two HIIT groups (both *p* > 0.05) suggesting a comparable long-term training response to both HIIT protocols. Analysis of circulating TGF-β1 mRNA levels in response to the training intervention revealed that TGF-β1 transcript levels at baseline were at a low level (in some participants at the lower detection limit) and were not induced by an acute exercise bout (*p* > 0.05; Supplemental Figure 2). After the 4-week training intervention, TGF-β1 mRNA levels were significantly increased in response to an acute high-intensity running bout (*p* < 0.001; Supplemental Figure 2). Correlation analysis to reveal miRNA and TGF-β1 mRNA dependent expression in response to the intervention revealed a good correlation between both miRNAs and TGF-β1 mRNA in both HIIT groups (all *r* ≥ 0.74, *p* ≤ 0.006; Figure 4).

### miR-222 and -29c levels during incremental continuous running

**Figure 5:**
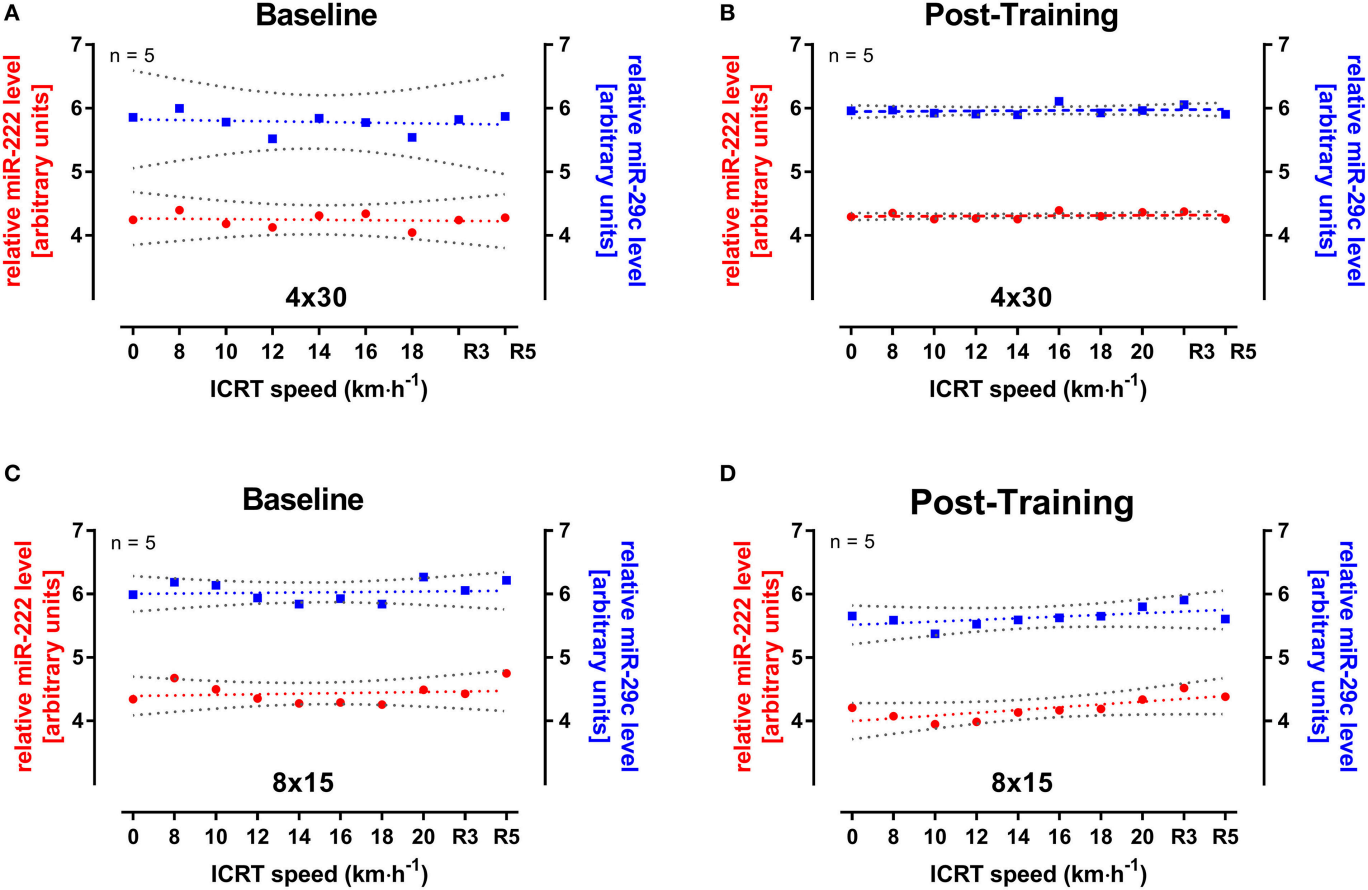
miR-222 and miR-29c levels during incremental continuous running test (ICRT) at baseline and follow-up. Five participants from either HIIT group were randomly selected and miRNA levels were determined during ICRT at **(A,C)** baseline and **(B,D)** follow-up. No significant change in miR-222 (red) or-29c (blue) levels was detected during ICRT or subsequent recovery (R3, R5). At follow-up, variation of miRNAs levels was reduced in the 4 × 30 HIIT group indicated by decreased widths of 95% CIs (gray) but not in the 8 × 15 HIIT group. Data are represented as mean with linear regression and 95% CI. R3, after 3 min of rest; R5, after 5 min of rest.

Since the elevation of miRNA levels might depend on exercise intensity or duration, miR-222 and -29c were also analyzed in five male participants from both HIIT groups during continuous runs at baseline and follow-up ICRT. All selected subjects reached ~18 km·h^−1^ which equaled a test duration of 20–24 min. Interestingly, no change in miRNA levels was seen for the analyzed subjects during or after the test in both groups (Figure 5). However, the range of miRNA levels over the entire 45 samples of the 4 × 30 HIIT group was altered between baseline and follow-up exercise test documented by reduced widths of 95% CIs (Figures 5A,B). For miR-222 in the 4 × 30 group, lower 95% CI = 4.017 and upper 95% CI = 4.469 changed to 4.274 and 4.338, respectively. For miR-29c in the 4 × 30 group, lower 95% CI = 5.366 and upper 95% CI = 6.198 changed to 5.905 and 6.016, respectively. This effect was not documented in the 8 × 15 HIIT group (Figures 5C,D). For miR-222 in the 8 × 15 group, lower 95% CI = 4.261 and upper 95 % CI = 4.596 changed to 4.036 and 4.349, respectively. For miR-29c in the 8 × 15 group, lower 95% CI = 5.869 and upper 95% CI = 6.177 changed to 5.467 and 5.796, respectively.

### miR-222 and HIIT response

**Figure 6:**
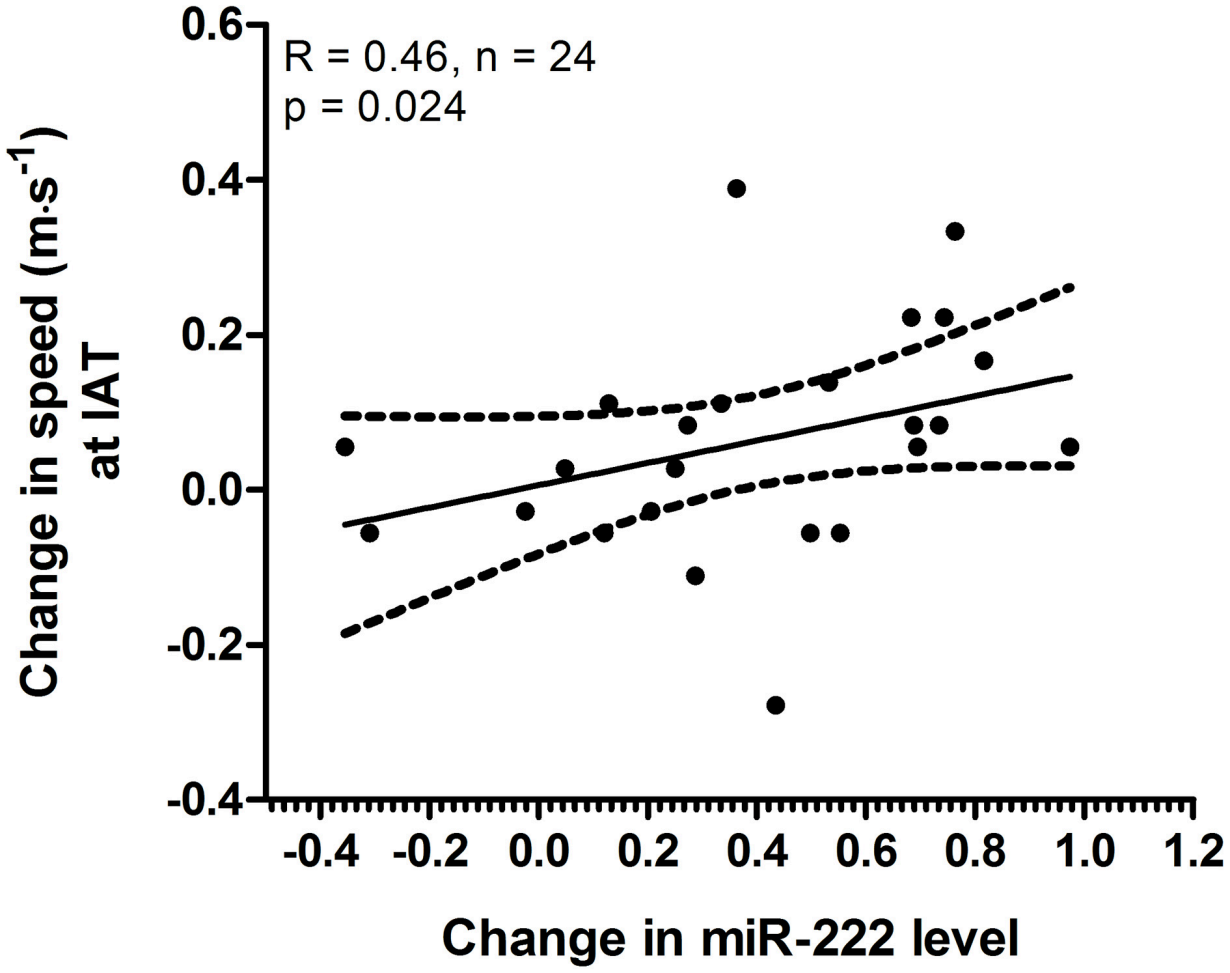
Acute changes in miR-222 at baseline were associated with increase in speed at individual anaerobic threshold (IAT). The analysis of acute changes of miR-222 levels within the 4 × 30 HIIT group at baseline showed a correlation with change in speed at IAT (baseline to follow-up). Individual data points are shown with 95% CI and linear regression.

Since we observed specific changes in speed at IAT for the 4 × 30 HIIT group and specific changes in acute miR-222 levels in this group, we analyzed if acute changes in miR-222 at baseline were associated with the overall training response in terms of increase in speed at IAT after the intervention. We found that a correlation existed between acute changes of miR-222 levels in response to a single exercise session at baseline and an improvement of running speed at IAT during follow-up extermination (*r* = 0.46, *p* = 0.024; Figure 6).

## Discussion

In this 4-week intervention study, we analyzed the effect of two workload-matched HIIT protocols on exercise parameters and circulating miR-222 and -29c levels in young healthy females and males. In brief, our main findings are (1) speed at IAT was increased by a 4 × 30 but not a 8 × 15 HIIT protocol, (2) miR-222 and -29c levels were elevated by a single session of 4 × 30 but not 8 × 15 HIIT at baseline, (3) miR-222 levels were still elevated by 4 × 30 HIIT at follow-up while again no effect was seen in the 8 × 15 HIIT group, (4) resting miR-222 and -29c levels were increased after the intervention in both HIIT groups and (5) miR-222 and -29c levels were not increased during incremental continuous runs.

Despite the fact that miRNAs have been identified as important modulators of exercise adaptations, only few studies have addressed changes in miRNA levels in response to training interventions (reviewed by Flowers et al., 2015; Polakovičová et al., 2016; Silva et al., 2017). Among other miRNAs regulated by physical activity, miR-222 and miR-29c are of particular interest as they may induce beneficial effects on the heart and the vasculature. In rodents, prolonged physical activity has been shown to induce cardiac miR-222 and -29c levels (Soci et al., 2011; Melo et al., 2014; Liu et al., 2015). Liu et al. (2015) investigated the mechanisms involved in cardiovascular adaptations in mice subjected to either wheel running or swimming exercise and found that miR-222 was necessary for exercise-induced growth of cardiomyocytes and thus for physiological hypertrophy. In humans, Baggish et al. (2011) originally detected acute as well as resting circulating miR-222 to be elevated in competitive male rowing athletes after 90 d of training (1–3 h/session, open-water/ indoor rowing). Later, a retrospective study of young males suggested miR-222 as useful biomarker of exercise mode-specific training adaptations since resting plasma levels of miR-222 were significantly elevated in endurance athletes compared to controls (Wardle et al., 2015). miR-29c, which belongs to the miR-29 family including−29a,−29b-1/2, and -29c transcribed from an identical pri-miR, has also been suggested to be regulated by exercise and to affect cardiac adaptions in rodents (van Rooij et al., 2008; Soci et al., 2011; Melo et al., 2014). Moreover, studies on myocardial infarcted rats subjected to a 10-week swimming protocol showed that reduced collagen deposition in the remote myocardium and in the border zone of the infarcted region was associated to miR-29 functions (Melo et al., 2014). In humans, one study reported miR-29c to be elevated in natural killer (NK) cells of healthy males after cycle ergometry at ~75% VO_2max_ (Radom-Aizik et al., 2013). In direct comparison, our results suggest that a single all-out exercise session may be sufficient to induce an acute elevation in circulating miR-222 and -29c levels. In addition, HIIT may also cause increased resting levels of both miRNAs. Interestingly, we only observed an acute miRNA increase in the 4 × 30 HIIT group, while no effect was detected in the 8 × 15 HIIT group, neither at baseline nor at follow-up. Thus, the duration of the stimulus or the length of the recovery phase seems to be an important component for miRNA elevation. Moreover, levels of both miRNAs remained unchanged during baseline and follow-up ICRT, which suggests that an acute increase in circulating miR-222 and -29c levels depends on physiological changes specifically associated with HIIT. To this respect, hypoxic stress which has been observed during HIIT (Abe et al., 2015) has been reported to trigger elevated cellular miR-222 levels (Camps et al., 2014; Xu et al., 2017). Furthermore, miR-222 is one of the most abundant miRNAs in exosomes released from ischemic cardiomyocytes (Ribeiro-Rodrigues et al., 2017). Besides hypoxic signaling, shear stress might be an important physiological stimulus during HIIT since miR-29c was reported as the top upregulated miRNA in human umbilical endothelial cells (HUVECs) under shear stress *in vitro* (Qin et al., 2010). However, the exact mechanisms of selected miRNA release into the blood stream and guided exosomal concentration of miRNAs especially in response to physical exercise is largely unknown (Chen et al., 2012; Makarova et al., 2014; Zhang et al., 2016). Thus, further studies will be needed to investigate whether the 4 × 30 HIIT protocol induces increased hypoxic stress and/ or shear stress in comparison to the 8 × 15 HIIT protocol.

The long-term effect on miRNA levels in our study was identical in that both groups presented an increase in miR-222 and -29c resting levels after the intervention, which for miR-222 is in line with the observation in rowing athletes after 90 d of training (Baggish et al., 2011). Further studies investigating the long-term effects of HIIT on miR-222 and -29c are missing from the literature which limits the discussion of the increased resting levels. However, our findings are interesting in the light of a an array-based miRNA comparison, which suggested that no difference in resting blood miRNA levels might exists between elite endurance athletes and matched moderately active controls (Backes et al., 2014). While this report contradicts the observation by Wardle et al. (2015) that miR-222 levels were significantly elevated in endurance athletes compared to matched controls, it might also indicate that the specific training modality (i.e., HIIT vs. continuous training) performed by the tested athlete has to be taken into account when comparing miRNA levels. To this respect, future studies should also investigate for how long resting miRNA levels remain elevated after a defined HIIT intervention. Our finding that acute differences between the HIIT groups existed but the long-term effect on resting levels was comparable might indicate that an additional mechanism for the constitutive expression of both miRNAs exists which results in an increased pool of concentrated miRNAs to be secreted. To this respect, both miRNAs have been suggested to be involved in regulation of the TGF-β pathway (Rao et al., 2011; Zhang et al., 2016) and our results showed a good correlation between both miRNAs and TGF-β1 mRNA levels. While this observation does not allow for the assumption that TGF-β1 is itself a target for miR-222 or-29c it suggests a common regulation of both miRNAs and components of the TGF-β pathway. These observations are partly in line with a report on exercise-induced circulating levels of Vascular Endothelial Growth Factor (VEGF) mRNA and miR-16 which is a known VEGF suppressor (Kilian et al., 2016). Even if the increase in circulating mRNA is not completely understood, it has been shown that extracellular vesicles transport several bioactive molecules including lipids, proteins, DNA, mRNAs, and miRNAs, which vary depending on the origin and stimulus and may serve to target recipient cells for reprogramming (Aliotta et al., 2010; de Jong et al., 2012; Hergenreider et al., 2012; Quesenberry et al., 2015). Since miR-222 and -29c are involved in cardiac adaptations in rodents (van Rooij et al., 2008; Liu et al., 2015) and TGF-β signaling plays a central role in the regulation of hypertrophic processes (Rao et al., 2011; Wang and Yang, 2012), elevated circulating levels of both miRNAs and TGF-β1 mRNA may be early markers of cardiac exercise hypertrophy.

The cellular origin of circulating miR-222 and -29c and mechanisms involved in their expression regulation are still under investigation. Both miRNAs are highly expressed in human endothelial cells (ECs) and EC-derived exosomes (Suárez et al., 2007; Qin et al., 2010; van Balkom et al., 2015). Another quantitative source of circulating miRNAs might be the erythrocyte population. However, a look-up in the human adult short RNA transcriptome (Doss et al., 2015) reveled that miR-222 and -29c are expressed at very low rates (0.0022% and 1.65 × 10^−4^%, respectively, compared to miR-486-5p) in erythrocytes. miR-222 and -29c are also not among the most abundant miRNAs in peripheral blood mononuclear cells (PBMC) and a look-up of original data (Vaz et al., 2010) revealed, that miR-222 and -29c were expressed at a mean of 0.4% and 2.6% compared to miR-let-7a. Furthermore, miR-222 and -29c are not highly abundant in resting NK cells (Wang et al., 2012) or neutrophils (Nelson et al., 2007). The group of Cooper and colleagues (Radom-Aizik et al., 2010, 2012) suggested that neither miR-222 nor-29c was significantly altered by exercise in PBMCs nor in neutrophils but found miR-29c upregulated in NK cells after cycling at ~75% VO_2max_ without a change for miR-222. By contrast, it has also been reported that serum miRNAs can be increased after myocardial damage (Kuwabara et al., 2011) and that miR-222 is one of the most abundant miRNAs in cardiomyocyte ischemic exosomes (Ribeiro-Rodrigues et al., 2017). This finding is in line with observations from animal studies reporting on elevated cardiac miR-222 levels in mice subjected to exercise without elevation in skeletal muscle (Liu et al., 2015). Thus, it seems conceivable that ECs and cardiomyocytes are the main source for elevated miR-222 and -29c levels during HIIT, while NK cells might contribute to some extent.

While we detected an effect on maximal speed > 2.5% in both HIIT groups, we observed a significant improvement of speed at IAT +3.6% only for the 4 × 30 HIIT group. In terms of peak power, two early studies on HIIT reported comparable effects. Jansson et al. (1990) also analyzed all-out HIIT in moderately trained subjects (10 sessions, 4–6 weeks, 3 × 30 s on cycle ergometer) and found a mean 2.4% increase in peak power output. Another study including untrained males and all-out HIIT (21 sessions, 7 weeks, 4–10 × 30 s on cycle ergometer) reported a mean 2.3% increase in peak power output (McKenna et al., 1993). Interestingly, both studies applied very different rest durations of 15–20 min and 30 s–4 min, respectively, suggesting that peak power is not strongly affected by HIIT rest duration. Improvement of speed at IAT has also been reported in other HIIT studies including Gojanovic et al. (2015) who reported a +5.6% increase in speed at IAT in trained runners (8 sessions, 4 weeks, 4–5 × 60% of time to exhaustion at vVO_2max_ on treadmill). While the optimal HIIT modalities in terms of intensity, work rest-rest ratio and rest duration are still under investigation (Sloth et al., 2013; Weston et al., 2014; Milanović et al., 2015), our data suggest that a 8 × 15 HIIT protocol with short work/ rest durations is not effective to improve speed at IAT.

Correlation analysis of the 4 × 30 HIIT group also suggested that acute changes in miR-222 were associated with an improvement of speed at IAT while both effects were not observed in the 8 × 15 HIIT group. Recent studies have already suggested that changes in HIIT work/rest ratios could induce different physiological adaptations (Kavaliauskas et al., 2015; Cipryan et al., 2016; Islam et al., 2017). With respect to the reported functions of miR-222 on cardiac hypertrophy it is interesting to speculate that heart muscle economy is improved as a response to a specific HIIT protocol and that these adaptations are indicated by changes of miR-222 levels as a functional marker. It has also been suggested that miR-222 affects cardiac angiogenesis implicated in healthy hypertrophy (Ribeiro-Rodrigues et al., 2017) which might lead to improved oxygen transport to the heart and could thus further improve heart muscle work. We also detected a strong effect on acute HR recovery of > 24% improvement for both HIIT groups. This observation confirms a recent observation by Stöggl and Björklund (2017) who reported that HR recovery of even endurance athletes could be improved by >11% (27 exercise sessions, 4 × 4 min at > 90% HR_max_). While these observation might indicate cardiovascular training adaptations in terms of increased stroke volume, it has been suggested that HR recovery following exercise testing is a measure for cardiac autonomic function and that a slow HR decline indicates reduced parasympathetic reactivation (Okutucu et al., 2011; Stöggl and Björklund, 2017). To this respect, the inert nature of HIIT with rapid changes between work and rest has been discussed to strongly affect the autonomic nervous system (Stöggl and Björklund, 2017), while HIIT work/rest duration do not seem to strongly affect cardiac autonomic function (Cipryan et al., 2016).

## Conclusions

We conclude that HIIT can induce increased miR-222 and miR-29c levels in healthy females and males. Compared to a 8 × 15 HIIT protocol, a workload-matched 4 × 30 HIIT protocol also induced acute miR-222 and miR-29c elevations at baseline, which were associated with improved power output at IAT at follow-up. Thus, miR-222 and miR-29c may help to monitor HIIT response in general and to identify optimal work/rest combinations. Moreover, miR-222 and miR-29c could represent functional markers for the optimization of cardioprotective HIIT regimes in primary and secondary prevention. Further research is needed to show if HIIT-induced miR-222 and -29c levels are associated with structural changes of the heart.

### Limitations

First, our findings may not be directly translated to other groups or populations as our study involved young healthy female and male Caucasians. Even if HIIT has also been proposed for patients with lifestyle-induced chronic diseases such as coronary artery disease or heart failure it may be detrimental for cardiac insulin sensitivity and blood flow capacity as shown for healthy but untrained middle-aged men (Eskelinen et al., 2016). Second, the current results are based on the determination of circulating miRNAs from whole blood and the white blood cell number has not been determined. Even though only NK cells seem to be a relevant source of miR-222 and -29c, this limitation might have affected our results to some extent. Furthermore, as miR-222 and miR-29c might be concentrated in MVs or associated to carrier proteins with the potential to shuttle into target cells, future studies should address the effect of physical exercise on miRNA in different blood components. Future studies are also needed to investigate if exercise-induced changes in circulating miR-222 and -29c levels are associated with cardiac adaptations in humans. Levels of circulating miRNAs were calculated using spike-in control as described (Fichtlscherer et al., 2010; Schlosser et al., 2015; Schmitz et al., 2017b) which is limited in the control of pre-analytical variations. However, as the performed sample preparation procedure had a high level of standardization in our setting, pre-analytical variations were reduced to a minimum. The sample size of our training groups might also be some limitation and future studies involving larger groups and longer observation periods post-exercise may generate additional insight. Although all HIIT sessions were controlled, we cannot completely exclude effects of extra non-prescribed training.

## Author contributions

BS designed and coordinated the study, enrolled and tested participants, analyzed and interpreted the data, and drafted the manuscript. FR enrolled and tested participants, collected samples, performed sample preparation and miRNA measurements, analyzed and interpreted data, and drafted the manuscript. KS and MM participated in sample collection. LT tested participants and analyzed training and testing data. AK coordinated the study, enrolled and tested participants. MK and S-MB interpreted data and helped finalizing the manuscript. All authors read and approved the final version of the manuscript.

### Conflict of interest statement

The authors declare that the research was conducted in the absence of any commercial or financial relationships that could be construed as a potential conflict of interest.
