# Supplementary material for: Longer Work/Rest Intervals During High-Intensity Interval Training (HIIT) Lead to Elevated Levels of miR-222 and miR-29c
Source: Front Physiol. 2018 Apr 17;9:395. doi: 10.3389/fphys.2018.00395 (PMC5913345; doi:10.3389/fphys.2018.00395)
Supplement: Supplementary file 1 [file DataSheet1.PDF]

Supplemental Material

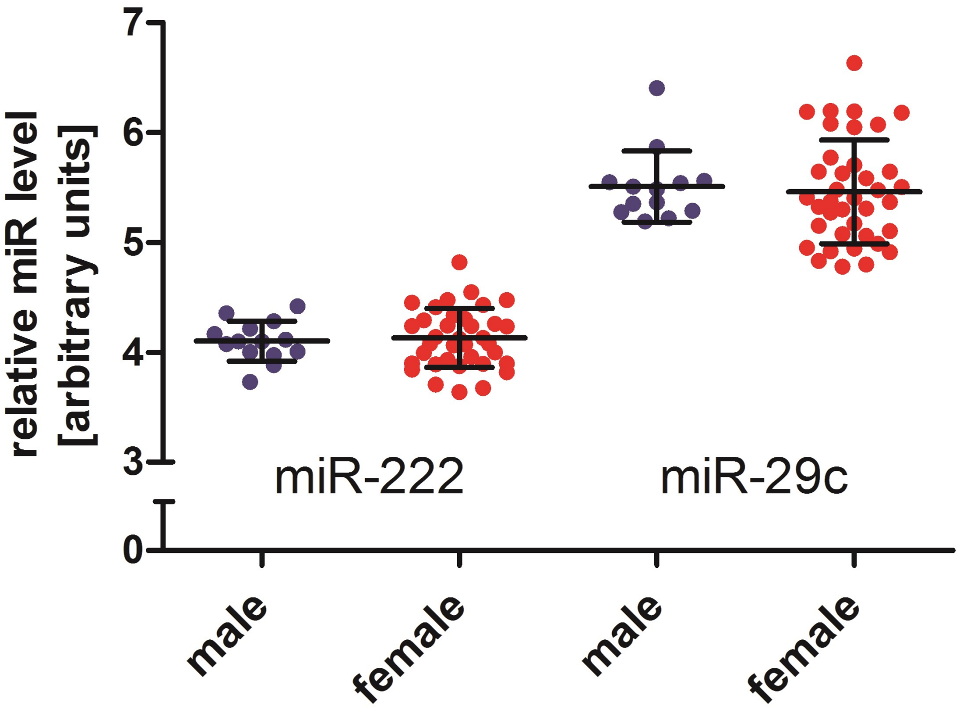

Supplemental figure 1:

Comparison of female and male microRNA-222-3p and -29c-3p levels at baseline. No significant difference was detected in dependence of sex.

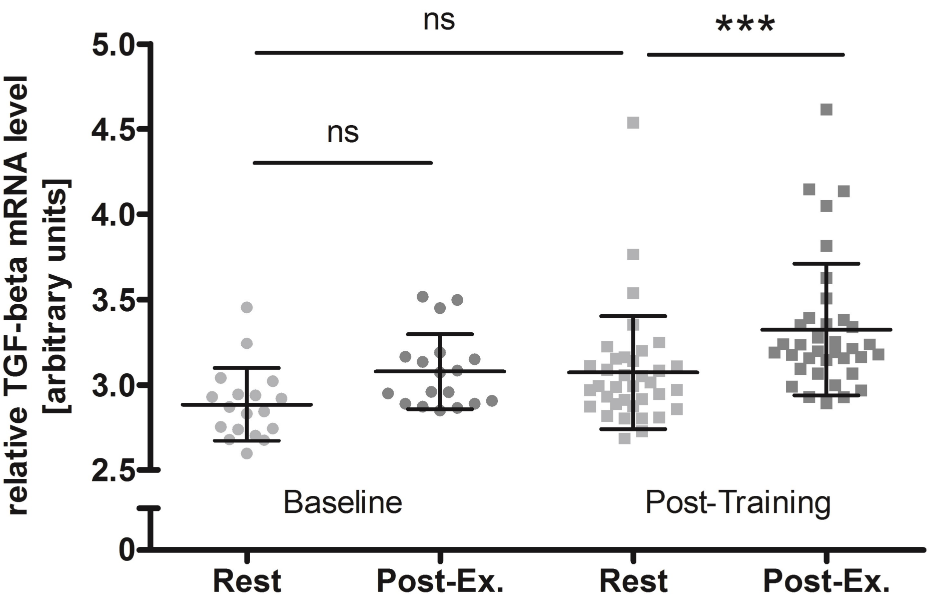

Supplemental figure 2:

Resting and post-exercise transforming growth factor-beta 1 (TGF-β1) mRNA levels at baseline and after 4 weeks of high-intensity interval training (HIIT). Circulating TGF-β1 mRNA levels were significantly elevated during exercise after the training intervention. Data was available for 18 participants at baseline and 36 participants post-training since TGF-β1 transcript levels were at the lower detection limit primarily at baseline. ***$p < 0.001$, ns = not significant.
